# Impact of high-flow oxygen therapy during exercise in idiopathic pulmonary fibrosis: a pilot crossover clinical trial

**DOI:** 10.1186/s12890-021-01727-9

**Published:** 2021-11-08

**Authors:** Diana Badenes-Bonet, Pilar Cejudo, Anna Rodó-Pin, Clara Martín-Ontiyuelo, Roberto Chalela, Jose Antonio Rodríguez-Portal, Rosa Vázquez-Sánchez, Joaquim Gea, Xavier Duran, Oswaldo Antonio Caguana, Diego Agustín Rodriguez-Chiaradia, Eva Balcells

**Affiliations:** 1 Respiratory Department, Servei de Pneumologia, Hospital del Mar, Passeig Marítim 27, 08003 Barcelona, Spain; 2 Pompeu Fabra University (UPF), Barcelona, Spain; 3 Grupo CB06/06/0043, Centro de Investigación en Red de Enfermedades Respiratorias, (CIBERES), Instituto de Salud Carlos III (ISCIII), Barcelona, Spain; 4 Grupo CB17/06/00030, Centro de Investigación en Red de Enfermedades Respiratorias, (CIBERES), Instituto de Salud Carlos III (ISCIII), Sevilla, Spain; 5 IMIM (Hospital del Mar Medical Research Institute), Barcelona, Spain; 6 Unidad Médico-Quirúrgica de Enfermedades Respiratorias, University Hospital Virgen del Rocío, Sevilla, Spain; 7 Biomedical Institute of Seville (IBIS), Sevilla, Spain; 8 Scientific, Statistics and Technical Department, Hospital del Mar-IMIM, Barcelona, Spain

**Keywords:** Idiopathic pulmonary fibrosis, High-flow nasal cannula, Oxygen therapy, Exertional desaturation

## Abstract

**Background:**

Supplemental oxygen delivered with standard oxygen therapy (SOT) improves exercise capacity in patients with idiopathic pulmonary fibrosis (IPF). Although high-flow nasal cannula oxygen therapy (HFNC) improves oxygenation in other respiratory diseases, its impact on exercise performance has never been evaluated in IPF patients. We hypothesized that HFNC may improve exercise capacity in IPF subjects compared to SOT.

**Methods:**

This was a prospective, crossover, pilot randomized trial that compared both oxygenation methods during a constant submaximal cardiopulmonary exercise test (CPET) in IPF patients with exertional oxygen saturation (SpO_2_) ≤ 85% in the 6-min walking test. The primary outcome was endurance time (Tlim). Secondary outcomes were muscle oxygen saturation (StO_2_) and respiratory and leg symptoms.

**Results:**

Ten IPF patients [71.7 (6) years old, 90% males] were included. FVC and DL_CO_ were 58 ± 11% and 31 ± 13% pred. respectively. Tlim during CPET was significantly greater using HFNC compared to SOT [494 ± 173 vs. 381 ± 137 s, *p* = 0.01]. HFNC also associated with a higher increase in inspiratory capacity (IC) [19.4 ± 14.2 vs. 7.1 ± 8.9%, respectively; *p* = 0.04], and a similar trend was observed in StO_2_ during exercise. No differences were found in respiratory or leg symptoms between the two oxygen devices.

**Conclusions:**

This is the first study demonstrating that HFNC oxygen therapy improves exercise tolerance better than SOT in IPF patients with exertional desaturation. This might be explained by changes in ventilatory mechanics and muscle oxygenation. Further and larger studies are needed to confirm the benefits of HFNC in IPF patients and its potential usefulness in rehabilitation programs.

**Supplementary Information:**

The online version contains supplementary material available at 10.1186/s12890-021-01727-9.

## Background

Idiopathic Pulmonary Fibrosis (IPF) is a progressive, chronic and fibrosing interstitial pneumonia with a poor prognosis [1]. IPF is clinically characterized by exertional dyspnea and hypoxemia, which is secondary to V_A_/Q mismatching and diffusing capacity limitation, and markedly worsens during exercise due mainly to the latter mechanism [2]. However, other factors including ventilatory inefficiency and peripheral muscle dysfunction can also contribute to exercise limitation in these patients [3]. Moreover, as a consequence of the associated restrictive ventilatory pattern, patients present a rapid and shallow breathing that becomes even more evident during exercise. This leads to a high minute ventilation at the expense of an elevated respiratory rate, with a progressive reduced capacity to increase tidal volume (V_T_), reflected by a low inspiratory capacity (IC) [4].

Although some small studies have demonstrated the presence of muscle dysfunction in IPF patients [5, 6] its causes are still poorly understood. As it occurs in chronic obstructive pulmonary disease (COPD) they probably would include physical inactivity due to exertional dyspnea, malnutrition, poor oxygen delivery, drug-induced myopathy and ageing [7]. In this regard, Wickerson et al. [8] recently demonstrated a clear muscle deoxygenation in peripheral muscles of IPF patients even at low workloads. However, oxygen therapy using standard delivery devices (nasal cannula, Venturi masks) (SOT) has recently been demonstrated to improve exercise capacity, increasing endurance time, reducing blood oxygen desaturation and dyspnea and even improving skeletal muscle metabolism in IPF patients [9, 10]. Nevertheless, it is broadly recognized in clinical practice that SOT has important limitations to achieving optimal levels of oxygenation in such patients during exercise [11]. It is worth noting, however, that the use of high-flow nasal cannula (HFNC) has become common in recent years for the treatment of patients with acute non-hypercapnic respiratory failure [12]. This system can deliver heated and humidified oxygen at high flow rates (up to 60 L/min), achieving high inspiratory fractions of oxygen (FIO_2_) [12], and producing higher physiological benefits compared with SOT devices [13]. Moreover, in subjects with stable COPD and severe ventilatory limitation for instance, the use of HFNC can better improve endurance time and blood oxygen saturation during exercise than SOT [14]. In contrast, these higher benefits did not appear to be present in a previous study that compared HFNC and SOT in an heterogeneous group that included different interstitial lung diseases [15]. However, there is a complete lack of previous studies assessing the potential advantages of HFNC in IPF in particular. Another aspect that has not been evaluated to date is the impact of HFNC on skeletal muscle oxygenation, and its relationship with potential improvements in exercise tolerance in IPF patients. Near-infrared spectroscopy (NIRS) has emerged in the last decade as a non-invasive method to assess skeletal muscle blood flow and oxygenation, thus approaching this tissue metabolic status in several respiratory diseases [16], including IPF [8]. We hypothesized that HFNC oxygen therapy could be more efficient than SOT in improving exercise tolerance in IPF patients with exertional desaturation. Therefore, we designed a crossover trial with the objective of comparing the effect of these two oxygen supplementation methods on endurance time (Tlim) in patients with stable IPF. Secondary end-points were peripheral muscle oxygen saturation (StO_2_) during exercise as well as dyspnea and leg symptoms during exertion.

## Methods

### Study participants

Patients with IPF diagnosis, according to the 2018 international consensus guidelines [1], were consecutively recruited from specialized Interstitial Lung Disease (ILD) clinics in two tertiary teaching hospitals. Eligible patients were those reaching a mean pulse oximeter oxygen saturation (SpO_2_) ≤ 85% (WirstOx2 TM Model 3150 Oximeter. Nonin Medical, INC. Plymouth, MN, USA) during the 6-min walking test (6MWT) performed at room air [17]. Exclusion criteria were fibrotic ILD other than IPF, coexistence of COPD, asthma or moderate-to-severe pulmonary hypertension [18], and inability to perform a cardiopulmonary exercise test (CPET) due to osteo-articular or cognitive limitations.

### Study design

**Fig. 1:**
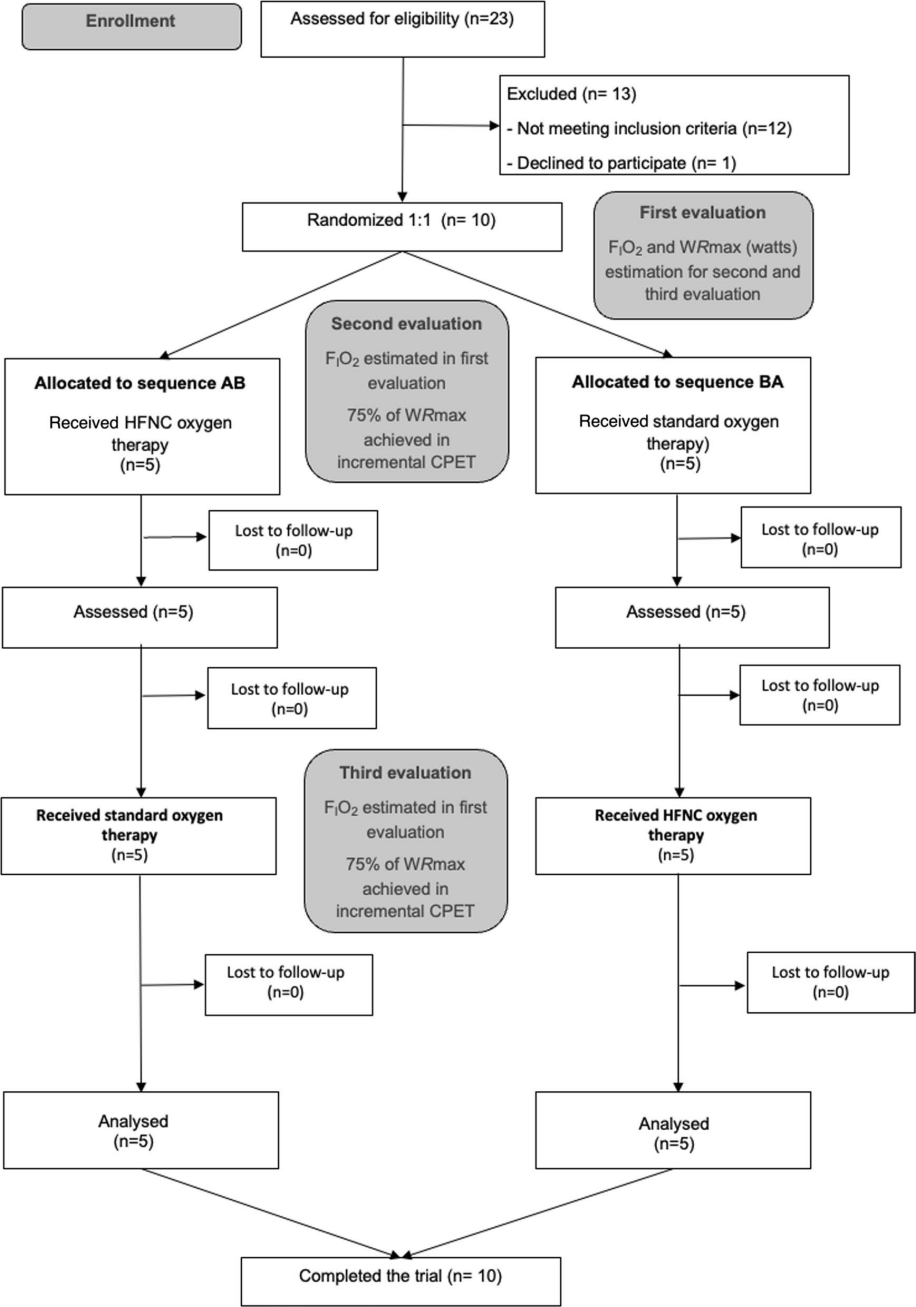
Study flow diagram. *IPF* idiopathic pulmonary fibrosis, *CPET* cardiopulmonary exercise test, *F*_*I*_*O*_*2*_ oxygen inspiratory fraction, *HFNC* high-flow nasal cannula, *WRmax* maximum work rate

This was a randomized crossover clinical trial conducted in three visits (Fig. 1). Patients were included from March 2019 to February 2020 and were subsequently randomized. The study was approved by the local ethics committee, carried out according to the principles of the Declaration of Helsinki for human investigations and registered as a clinical trial (NCT04564664). All participants signed the appropriate informed consent prior to their inclusion.

An initial screening visit was performed according to the study protocol. Sociodemographic and clinical variables were also collected including GAP (Gender-Age-Physiology) and body mass (BMI) indices, individual comorbidities, Charlson comorbidity index and treatments received. Conventional pulmonary function tests and the 6MWT were performed according to international guidelines [17, 19, 20]. In addition, quadriceps and hand-grip strength were also measured following the previously described methodology [21], and the highest value of at least three correct maneuvers was chosen for both measurements.

#### First exercise evaluation

To assess maximum exercise capacity (W*R*max) and to determine the FIO_2_ (Venturi mask) necessary to always maintain a SpO_2_ > 85%, a symptom-limited incremental CPET was performed by all patients. Subjects were randomized consecutively with a 1:1 allocation sequence to perform the first CPET with either HFNC or SOT.

#### Second and third evaluation

Following randomization all individuals performed two consecutive, submaximal CPET (75% of their W*R*max) with at least 24 h apart. Half of them started with the HFNC O_2_ supplementation and the remaining with SOT.

### Definitions and variables

The above mentioned three CPET were performed using a cycloergometer (Ergoline Medical Graphics Corporation, St. Paul, MN, USA) and a gas analyzer (Cardiorespiratory Diagnostic System, Ultimaseries TM, MediGraphics, Orlando, FL, USA). The tests were interrupted if adverse effects such as chest pain, changes in the electrocardiographic record or desaturation reaching SpO_2_ < 80% appeared despite oxygen supplementation.

#### Symptom-limited incremental CPET

This was performed to evaluate the patient’s W*R*max according to the ATS/ACCP standardization statement [22]. In this regard, a conventional increasing protocol (10 Watts/min) was used and subjects had to maintain a 50–60 rpm constant speed at all time. As previously mentioned, the appropriate FIO_2_ needed to maintain a SpO_2_ > 85% was obtained.

#### Submaximal CPET with SOT

This was performed using a conventional Venturi system at the FIO_2_ chosen in the incremental CPET.

#### Submaximal CPET with HFNC

This exercise test was also carried out at the FIO_2_ obtained in the incremental CPET using an AIRVO2 device in this case (Optiflow TM, Fisher and Paykel, New Zealand), with flows ranging between 40 and 60 l/min, heat and humidification. The endurance time (Tlim) for the latter two tests was defined as the point at which the patient was unable to maintain 60 rpm speed despite very active encouragement and was measured in seconds.

#### Vital and ventilation variables during all CPETs

SpO_2_ and heart rate (HR) were monitored with a pulse oximeter (Biox, OHMEDA, Madison, WI, USA) and blood pressure was assessed every 2 min. In addition, respiratory rate (RR) and tidal volume (VT) were recorded from the above mentioned exercise system. Inspiratory capacity (IC) maneuvers were carried out at the beginning and immediately following the end of the tests. The same was done for dyspnea and leg fatigue assessment through Borg scales.

#### Leg muscle saturation (StO_2_)

Leg muscle saturation (StO_2_) was also monitored using NIRS, (NIRO-200NX, Hamamatsu, Japan) during all CPET procedures in 7 patients, and specific data obtained at rest, during free-pedaling, submaximal exercise and recovery were further analyzed. The system was calibrated before each study following manufacturer’s recommendations and the output frequency was 1 Hz in all cases, with the sensor being placed on the left quadriceps (vastus medialis) [16]. Two specific values were considered for the analysis of StO_2_ behavior. On the one hand, its mean value during submaximal exercise (75% W*R*max) and on the other, the isotime value at task failure. Moreover, two derived variables were also evaluated: exercise induced StO_2_ fall (i.e. Mean StO_2_ at submaximal exercise − StO_2_ at rest) and exercise unloading-induced rebound (i.e. StO_2_ in the recovery phase − mean value at submaximal exercise).

### Sample size estimation and statistical analysis

Based on previous studies, a sample size of at least 8 subjects was required to detect a mean difference in Tlim equal or higher than 33% between HFNC and SOT [23], considering a 20% dropout rate [15, 23, 24]. Categorical variables were expressed as frequencies and continuous variables as mean and standard deviation (SD) or median and 25, 75 percentiles (p25–p75). Paired t-test was used for comparisons between the two O_2_ supplementation methods in normally distributed variables, and Wilcoxon test for those with non-normal distribution. Correlation analysis was performed using Spearman correlation coefficient. A *p* value < 0.05 was always considered as statistically significant. The analysis was performed using the IBM SPSS statistics pack for Windows (Version 23.0, IBM Corp., Armonk, NY, USA).

## Results

### Participant characteristics

The study flow diagram is detailed in Fig. 1. A total of 10 patients were finally enrolled in our crossover trial from March 2019 to January 2020. No adverse events were registered during any of the CPET performances, and all patients completed the trial. Baseline characteristics are summarized in Table 1. Subjects walked an average of 436 m (around 90% pred.) and presented a mean SpO_2_ of 81% in the 6MWT. Mild pulmonary hypertension as approximated by echocardiography was present in 4 patients. Peripheral muscle strength was preserved. During the incremental CPET patients achieved a mean workload of 81 watts (64% pred.) and the mean FIO_2_ needed to maintain SpO_2_ over 85% was 0.3. Ventilatory and cardiocirculatory characteristics of this incremental CPET are summarized in the Additional file 1: Table S1. No significant differences were present between those patients randomized to initiate the submaximal CPET with HFNC and those who began with SOT.

**Table 1:** Baseline population characteristics

	Total population (n = 10)
General characteristics	
Gender, male; n (%)	9 (90)
Age, years; mean (SD)	71.7 (6)
Former smoker, n (%)	8 (80)
BMI, kg/m^2^; mean (SD)	28.5 (5)
GAP index, median (p25-p75)	3 (2–3)
Treatments, n (%)	
Antifibrotic therapy	9 (90)
Ambulatory oxygen therapy on exertion	8 (80)
Ambulatory 24-h oxygen therapy	4 (40)
Comorbidities, n (%)	
Hypertension	4 (40)
Dyslipidemia	4 (40)
Diabetes mellitus	3 (30)
Sleep apnea	2 (20)
Ischemic heart disease	2 (20)
Pulmonary emphysema	2 (20)
Pulmonary hypertension^a^	4 (40)
Charlson comorbidity index; mean (SD)	4.1 (1.2)
Pulmonary function tests, mean (SD)	
FEV_1_, % pred	62 (13)
FVC, % pred	58 (11)
DL_CO_, % pred	31 (13)
6-min walking test, mean (SD)	
Distance, m	436 (131)
Distance, % pred	90 (20)
Initial SpO_2_, %	91 (4)
Minimum SpO_2_, %	76 (7)
Mean SpO_2_, %	81 (4)
ΔSpO_2_, %^b^	− 18 (10)
Peripheral muscle strength, mean (SD)	
Quadriceps strength, kg	41 (14)
Quadriceps strength, % pred	109 (39)
Hand-grip, kg	30 (12)
Hand-grip, % pred	111 (30)

*BMI* body mass index, *GAP* gender age physiology, *FEV*_*1*_ expiratory flow in the first second, *FVC* forced vital capacity, *DL*_*CO*_ carbon monoxide diffusion capacity, *SpO*_*2*_ peripheral oxygen saturation

^a^Pulmonary hypertension was assessed by echocardiography (systolic pulmonary artery pressure > 35 mm Hg)

^b^ΔSpO_2_%, percentage of change between baseline and exercise values

### Primary and secondary outcomes

**Fig. 2:**
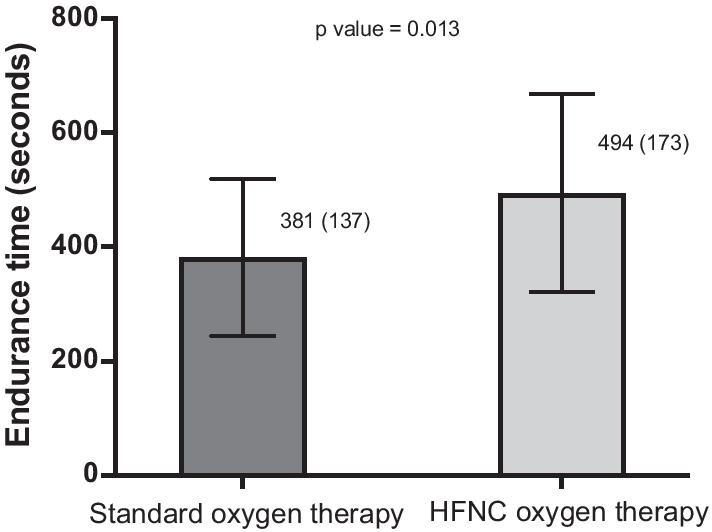
Endurance time (seconds) with standard oxygen therapy and HFNC during CPET. *HFNC* high-flow nasal cannula, *CPET* cardiopulmonary exercise test. Data are presented as mean and standard deviation

Table 2 compares most relevant parameters obtained between submaximal CPETs with HFNC or SOT. As stablished in the previous incremental CPET, F_I_O_2_ was 0.33 ± 0.07 for both supplementation methods. It is worth noting that Tlim was significantly greater (30%) during exercise with HFNC when compared with SOT (shown in Fig. 2). Differences in Tlim between both O_2_ supplementation methods inversely correlated with the mean SpO_2_ observed in the 6MWT (r = − 0.705, *p* = 0.02). Absolute differences between both supplementation methods in Tlim were also directly related to those observed in SpO_2_ at task failure in submaximal CPETs (r = 0.85, *p* = 0.002), showing a similar tendency with differences in mean StO_2_ obtained during submaximal exercise (r = 0.607, *p* = 0.148, respectively). No other correlations were found between Tlim and the remaining variables.

**Table 2:** Comparisons of CPET data with SOT and HFNC oxygen therapy

	SOT (n = 10)	HFNC (n = 10)	*p* value
Tlim (s)	381 (137)	494 (173)	**0.013**
O_2_ flow (l)			
Initial	8 (2.8)	40 (0)	**< 0.01**
End of test	9.1 (4.1)	45 (8.5)	**< 0.01**
VT (ml)			
Initial	710 (174)	646 (158)	0.20
End of test	1310 (466)	1333 (518)	1
Change (%)^a^	89 (27)	100 (54)	1
RR (rpm)			
Initial	27 (10)	26 (8)	0.16
End of test	44 (11)	43 (10)	0.87
VE (%pred.)			
Initial	27 (7)	25 (8)	0.17
End of test	81 (15)	78 (13)	0.46
IC (l)			
Initial	1.4 (0.5)	1.4 (0.5)	0.83
End of test	1.5 (0.6)	1.7 (0.6)	0.53
Change (%)^a^	7.1 (8.9)	19.4 (14.2)	**0.04**
HR (bpm)			
Initial	84 (16)	84 (16)	0.76
End of test	122 (17)	123 (20)	0.72
Isotime	118 (15)	118 (20)	0.92
HR (%pred.)			
Initial	56 (10)	56 (11)	0.80
End of test	82 (10)	82 (12)	0.77
SpO_2_ (%)			
Initial	97 (2)	97 (1)	0.72
End of test	90 (4)	90 (3)	1
StO_2_ (%)^b^			
Initial	45 (7.2)	47.1 (9.3)	0.35
End of test	43.4 (9.6)	47.2 (10.6)	0.12
Borg scale			
Initial			
Dyspnea	0.1 (0.3)	0.2 (0.4)	0.32
Leg fatigue	0 (0)	0.2 (0.6)	0.32
End of test			
Dyspnea	6.6 (1.8)	6.7 (2.4)	0.91
Leg fatigue	5.1 (3.4)	4.9 (3.1)	0.83

Each parameter is expressed as mean (standard deviation)

*CPET* constant work pulmonary exercise test, *SOT* standard oxygen therapy, *HFNC* high-flow nasal cannula, *Tlim* endurance time, *s* seconds, *O*_*2*_ oxygen, *VT* tidal volume, *RR* respiratory rate, *rpm* respirations per minute, *VE* pulmonary ventilation, *IC* inspiratory capacity, *HR* heart rate, *bpm* beats per minute, *SpO*_*2*_ peripheral oxygen saturation, *StO*_*2*_ peripheral muscle oxygen saturation

^a^Change (end of test value − initial value/initial value) * 100

^b^Measured in n = 7 subjects

**Fig. 3:**
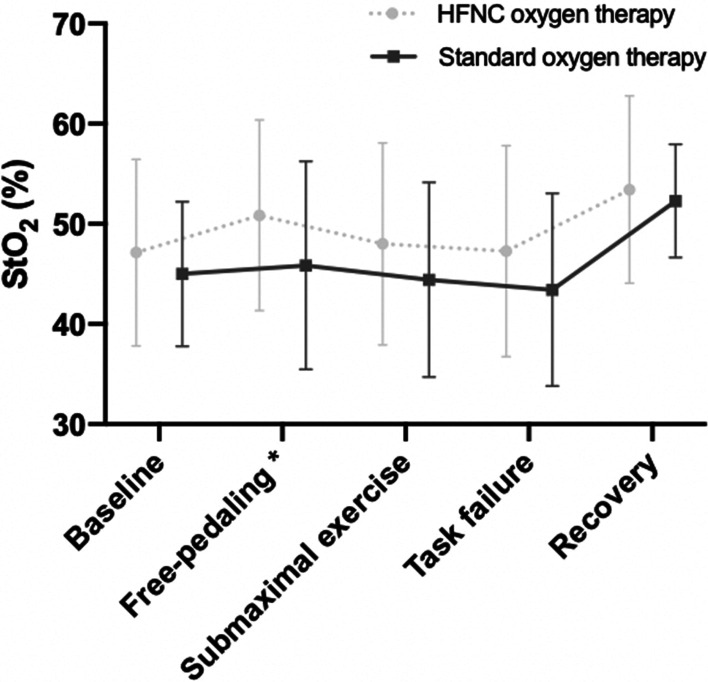
Peripheral muscle oxygen saturation (StO_2_) measured by NIRS during CPET performance with both oxygen devices (n = 7). *StO*_*2*_ muscle oxygen saturation, *NIRS* near-infrared spectroscopy device, *CPET* cardiopulmonary exercise test, *HFNC* high-flow nasal cannula*.* *p < 0.05. Data are presented as mean and standard deviation

In addition, a higher StO_2_ was observed with HFNC compared to SOT during free-pedaling exercise with a similar tendency for its mean value 75% W*R*max and isotime (shown in Fig. 3, and in the Additional file 2: Table S2). Moreover, although no changes were observed in the breathing pattern, a significantly higher percentage of improvement was observed in IC under HFNC if compared with SOT. Finally, no differences between the two oxygen devices were found in symptoms (either dyspnea or leg discomfort) at the end of the submaximal exercise test.

## Discussion

This prospective crossover trial is the first to demonstrate that HFNC oxygen therapy allows IPF patients to obtain a better exercise performance (i.e. higher Tlim) than SOT, probably to a better recruitment of alveolar spaces, reducing mechanical constraints and/or preventing early deoxygenation at muscle levels. Whatever the mechanisms are, HFNC appears as an excellent option to give oxygen supplementation during exercise to IPF patients.

There is only one previous paper, published by Suzuki et al., that approximates the usefulness of HFNC in ILD patients during exercise [15]. They also performed a randomized crossover study using HFNC versus SOT in a mixed pool of different fibrotic ILD patients who have in common that they reached a very low SpO_2_ (< 88%) during an incremental CPET. However, they found no differences among both methods in the whole group, probably due to its great heterogeneity [15]. Interestingly, when they performed a post-hoc analysis, they identified a subgroup of patients that were ‘better responders’ to HFNC than to SOT. Although the authors do not discuss the composition of this specific subgroup, the percentage of IPF was slightly higher than in the ‘non-responder group’. This last finding was crucial to lead us to designing the present study including only patients with IPF. Our results strongly support the notion that HFNC is a better option to give supplemental oxygen during exercise in this particular interstitial disease.

Different reasons should be considered to explain the better Tlim obtained with HFNC in the present study. First of all, as previously described and suggested in the Suzuki study, HFNC seems to increase the washout of physiological dead space, thus reducing ineffective ventilation and even improving the work of breathing [13, 15]. Secondly, HFNC can also produce a moderate increase in positive airway pressure that would prevent collapse of critical alveolar units [14]. Both complementary mechanisms are suggested in our study by the bigger improvement in IC with HFNC if compared with SOT, suggesting that the lung parenchyma of our IPF patients was still somewhat distensible and some additional alveolar units might have been recruited for more effective ventilation. The mechanism would be similar—although much less intense—than that previously observed using non-invasive respiratory support (i.e. BiPAP or CPAP) in IPF patients during exercise [25]. However, even with this theoretical improvement in alveolar ventilation, its impact on arterial blood oxygenation was absent since SpO_2_ during exercise was similar with both supplementation methods. Therefore, although somewhat speculative we hypothesize that one potential reason for the better exercise performance observed with HFNC was mostly related with an improvement in the mechanics of the respiratory system rather than to changes in pulmonary gas exchange. In contrast, the direct relationship observed in the present study between differences in Tlim and in task failure SpO_2_ between both submaximal exercises would indicate that oxygenation has probably played some role in the better improvement in exercise capacity observed with HFNC.

A complementary conclusion derives from the inverse correlation observed between the mean SpO_2_ values observed in the 6MWT and the amount of the differential benefits in Tlim obtained with HFNC oxygenation in the submaximal CPET. This might suggest that this last supplementation method would be especially indicated in those patients with greater exertional hypoxemia. A similar relationship between exertional SpO_2_ and exercise performance in CPET was observed in a previous study where supplemental oxygen was compared with placebo in IPF patients [9].

One novelty of the present study is the complementary assessment of peripheral muscle oxygenation during exercise in IPF patients. Interestingly, although no differences were observed in SpO_2_ during exercise among both oxygenation methods, our patients showed a significantly higher StO_2_ with HFNC than with SOT during free-pedaling and a tendency in the same direction during submaximal effort and at task failure (shown in Fig. 3). Moreover, differences in both SpO_2_ and StO_2_ among both oxygenation methods showed a trend to correlate with differences in Tlim. We observed no significant correlations between SpO_2_ and StO_2_ in any of the situations here analyzed. A finding that is in line with a previous study on ILD patients, where discrepancies were also observed between both variables during two incremental leg exercises [8], being explained by potential changes in muscle ability to extract and use the oxygen delivered by blood [8]. Muscle dysfunction has been suggested as a potential contributor to exercise intolerance in ILD patients [26], and more specifically, quadriceps strength has already been shown to be an independent factor to maximal exercise capacity (represented by VO_2_ peak) in IPF patients [6]. However, the mechanisms of muscle weakness in interstitial disorders still remain unclear, although as it occurs in other chronic lung diseases, physical deconditioning may play an important role [3]. In the present study we observed no loss in either upper or lower leg muscle strength (a property that mainly depends on muscle mass) but this does not necessarily exclude abnormalities in muscle endurance (more dependent on the aerobic metabolism). In fact, the absence of significant correlations between SpO_2_ and StO_2_ suggest this latter possibility.

With regard to symptoms, no differences were found in our study between neither dyspnea or leg fatigue at the end of the test between both oxygen supplementation methods. Previous investigations on ILD, or more specifically on IPF, have demonstrated less dyspnea and fatigue scores on exertion with SOT when compared with placebo [9, 27]. In the unique preceding study comparing HFNC with SOT, the authors were unable to find differences in symptoms [15].

Current guidelines recommend rehabilitation programs in ILD patients, as this has demonstrated clear benefits such as improvement in dyspnea, exercise capacity and quality of life [28, 29]. However, data are lacking on the effects of different methods of oxygen supplementation during training in patients with exertional desaturation. From our present results we could anticipate that HFNC could be a useful oxygen supplementation method to obtain better outcomes from rehabilitation programs and therefore an excellent alternative to improve functional capacity in IPF subjects. Given the limited access to HFNC oxygen devices, its use nowadays would be limited mainly to controlled training programs. However, the ongoing HOPE study, which has the objective of determining the effect of different oxygenation methods during exercise training in IPF patients treated with Nintedanib, will probably clarify this point [30].

One of the strengths of our study is that it focuses on IPF patients, reducing the heterogeneity derived from the inclusion of different fibrotic ILDs employed in previous studies. In fact, patients with IPF seem to present a higher alveolar-arterial O_2_ gradient (AaPO_2_) during exercise compared to other ILDs, indicating worse VA/Q relationships and/or O_2_ diffusion capacity [2]. This factor might explain the absence of clear conclusions on the use of HFNC in previous studies that included mixed ILDs. This the first study that compares both oxygen supplementation methods including the assessment of different components that can contribute to exercise limitation in IPF patients. There are also, however, some limitations that should be recognized. The study population was relatively small, and patients were not totally blind regarding the two supplementation techniques. Although they did not know either the flow or FIO_2_ supplied, they could easily identify the oxygenation method. However, this limitation is almost impossible to overcome since both devices generate clearly differentiated perceptions.

## Conclusions

Exercise intolerance is the predominant symptom in IPF. However, to date there are only few treatment strategies directed to improving exercise capacity in such patients. On the basis of the present results, we suggest that HFNC oxygen therapy is a better option than SOT for improving exercise capacity in those IPF patients with exertional desaturation. Taking all our results together we can speculate that reasons for the improvement observed in Tlim in our IPF patients were related to positive changes in ventilatory mechanics and perhaps in muscle oxygenation. However, further and larger studies are needed to confirm these benefits and to establish its appropriate use in rehabilitation programs.

## Supplementary Information

**Additional file 1: Table S1**. Incremental CPET parameters in the overall population.

**Additional file 2: Table S2**. Peripheral muscle oxygen saturation (StO_2_) measured by NIRS.

## Data Availability

The datasets supporting the conclusions of this article are included within the article and its additional files. Datasets will be available from the corresponding author on reasonable request.

## Abbreviations

6MWT: Six-minute walking test
AaPO_2_: Alveolar-arterial O_2_ gradient
BMI: Body mass index
COPD: Chronic obstructive pulmonary disease
CPET: Cardiopulmonary exercise test
DL_CO_: Carbon monoxide diffusion capacity
FEV_1_: Expiratory flow in the first second
F_I_O_2_: Inspiratory fraction of oxygen
FVC: Forced vital capacity
GAP: Gender-Age-Physiology
HFNC: High-flow nasal cannula
HR: Heart rate
IC: Inspiratory capacity
ILD: Interstitial lung disease
IPF: Idiopathic pulmonary fibrosis
NIRS: Near-infrared spectroscopy
RR: Respiratory rate
SD: Standard deviation
SOT: Standard oxygen therapy
SpO_2_: Peripheral oxygen saturation
StO_2_: Muscle oxygen saturation
Tlim: Endurance time
VE: Pulmonary ventilation
V_T_: Tidal volume
W*R*max: Maximum exercise capacity
