# Supplementary material for: Impact of high-flow oxygen therapy during exercise in idiopathic pulmonary fibrosis: a pilot crossover clinical trial
Source: BMC Pulm Med. 2021 Nov 8;21:355. doi: 10.1186/s12890-021-01727-9 (PMC8573951; doi:10.1186/s12890-021-01727-9)
Supplement: Supplementary file 1 — Additional file 1: Table S1. Incremental CPET parameters in the overall population. [file 12890_2021_1727_MOESM1_ESM.docx]

**Additional file 1. Incremental CPET parameters in the overall population**

|  | **Total population**  **(n=10)** |
| --- | --- |
| **Workload (watts)** | 81 (27) |
| **Workload (%pred.)** | 64 (15) |
| **FIO_2_ (%)**  **Initial**  **End of test** | 0.25 (0.04)  0.33 (0.7) |
| **SpO_2_ (%)**  **Initial**  **End of test** | 95 (2)  90 (4) |
| **VE (l/min)**  **Initial**  **End of test** | 16 (7)  54 (17) |
| **VE (% pred.)**  **Initial**  **End of test** | 23 (7)  78 (17) |
| **V_T_ (ml)**  **Initial**  **End of test** | 723 (224)  1273 (448) |
| **IC (l)**  **Initial**  **End of test** | 1.5 (0.4)  1.7 (0.4) |
| **RR (rpm)**  **Initial**  **End of test** | 23.4 (9.7)  44.3 (10) |
| **HR (bpm)**  **Initial**  **End of test** | 86 (17)  120 (19) |
| **HR (%pred.)**  **Initial**  **End of test** | 57 (12)  77 (11) |
| **Borg scale,**  **Initial**  **Dyspnea**  **Leg Fatigue**  **End of test**  **Dyspnea**  **Leg Fatigue** | 0.1 (0.3)  0.4 (1.3)  6 (2.4)  5 (2.7) |

*Abbreviations:* CPET, cardiopulmonary exercise test; FIO_2_, oxygen inspiratory fraction; SpO_2_, peripheral oxygen saturation; VE, pulmonary ventilation; V_T_, tidal volume; IC, inspiratory capacity; RR, respiratory rate; rpm, respirations per minute; HR, heart rate; bpm, beats per minute.

Data are presented as mean (standard deviation).
