# Supplementary material for: Impact of high-flow oxygen therapy during exercise in idiopathic pulmonary fibrosis: a pilot crossover clinical trial
Source: BMC Pulm Med. 2021 Nov 8;21:355. doi: 10.1186/s12890-021-01727-9 (PMC8573951; doi:10.1186/s12890-021-01727-9)
Supplement: Supplementary file 2 — Additional file 2: Table S2. Peripheral muscle oxygen saturation (StO2) measured by NIRS. [file 12890_2021_1727_MOESM2_ESM.docx]

**Additional file 2. Peripheral muscle oxygen saturation (StO_2_) measured by NIRS**

|  | **SOT**  **(n=7)** | **HFNC**  **(n=7)** | **p value** |
| --- | --- | --- | --- |
| Baseline | 45 (7.2) | 47.1 (9.3) | 0.345 |
| Free-pedaling | 45.8 (10.4) | 50.8 (9.5) | **0.046** |
| Submaximal exercise | 44.4 (9.7) | 48 (10.1) | 0.176 |
| Task failure (isotime) | 43.4 (9.6) | 47.2 (10.6) | 0.116 |
| Recovery | 52.3 (5.6) | 53.4 (9.3) | 0.528 |
| P2-P1 | -0.6 (5.4) | 0.9 (5.7) | 0.612 |
| P3-P2 | 7.8 (9.9) | 5.4 (6.9) | 0.233 |

*Abbreviations:* NIRS, near-infrared spectroscopy; SOT, standard oxygen therapy; HFNC, high-flow nasal cannula.

P1, baseline tissue oxygen saturation; P2, tissue oxygen saturation at 75% W*R*max; P3, recovery after exercise tissue oxygen saturation. P2-P1, exercise induced fall in StO_2_; P3-P2, exercise unloading-induced rebound in StO_2._

Each parameter is expressed as mean (standard deviation).
